# Intercellular transmission of cGAS-STING signaling in cancer

**DOI:** 10.20892/j.issn.2095-3941.2022.0750

**Published:** 2023-03-02

**Authors:** Qirou Wu, Xiaohong Leng, Pinglong Xu

**Affiliations:** 1 MOE Laboratory of Biosystems Homeostasis and Protection and Zhejiang Provincial Key Laboratory for Cancer Molecular Cell Biology, Life Sciences Institute, Zhejiang University, Hangzhou 310058, China; 2 Institute of Intelligent Medicine, Hangzhou Global Scientific and Technological Innovation Center, Zhejiang University (HIC-ZJU), Hangzhou 310058, China; 3 Cancer Center, Zhejiang University, Hangzhou 310058, China

Cellular communication is necessary for organizing multicellular organisms, and is critical in tumorigenesis and progression. Immune and tumor cells use the cGAS-STING mechanism to sense and respond to genomic instability, DNA damage, and mitochondrial dysfunction induced by extra- and intracellular stresses. As an essential component of innate nucleic acid sensing, cGAS-STING signaling is critical in various pathological processes, including microbial infection, autoimmunity, inflammation, organ degeneration, and malignancy. The cytosolic DNA sensor cyclic GMP-AMP synthase (cGAS) senses aberrant or displaced dsDNA in the cytosol and synthesizes 2′3′-cyclic GMP-AMP (cGAMP). Endoplasmic reticulum (ER)-resident adaptor stimulator of interferon genes (STING), mobilized by this second messenger, is trafficked among the ER, ER-Golgi intermediate compartment, and Golgi apparatus, and forms distinct STING signalosomes, which in turn trigger various molecular programs controlling mRNA translation, autophagy, organelle reorganization, and interferon (IFN) production^1^. The non-canonical functions of cGAS-STING signaling have recently gained extensive attention. These functions occur in various cellular processes, including senescence, autophagy, and cap-dependent mRNA translation^1,2^.

Notably, the complexity of cGAS-STING signaling is further enhanced by an intriguing feature of intercellular transmission (**Figure 1**) that has recently been established in several seminal discoveries. The effective intercellular transfer of cGAS-STING signaling enables the sharing of critical information arising from infected, injured, or cancerous cells and the activation of multiple bystander cells in innate immune responses. In cancers, intercellular communication of cGAS-STING signaling connects the tumor microenvironment and pathogenic, immune, and stromal cells, thus promoting or compromising various immune responses and substantially altering disease progression. Understanding this intercellular interplay would greatly aid in developing therapeutics against malignancy. In this perspective, we discuss current knowledge, effects on cancer biology, and implications of in cancer progression and therapeutics.

**Figure 1:**
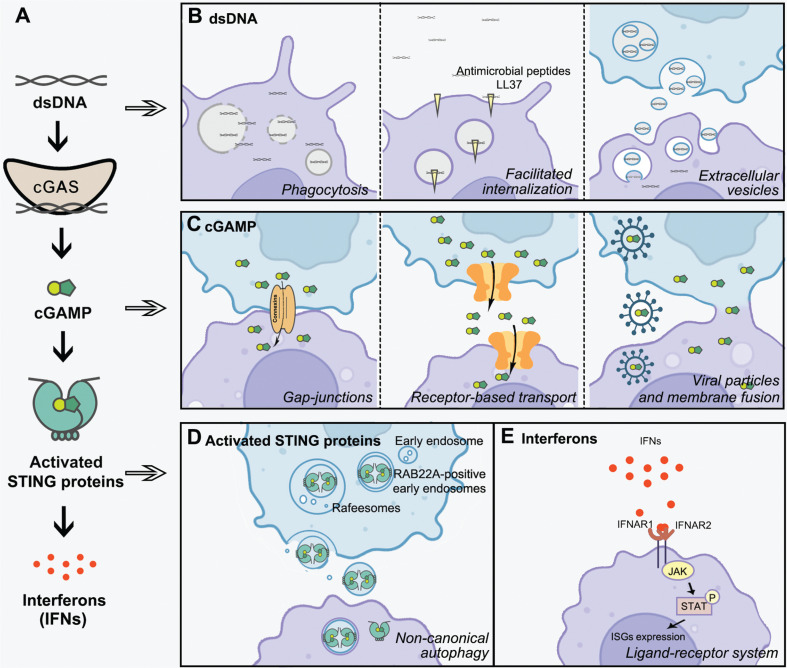
Intercellular communication in cGAS-STING signaling. (A) Schematic diagram of canonical cGAS-STING signaling. The DNA sensor cyclic GMP-AMP synthase (cGAS) recognizes a wide range of double-strand DNA (dsDNA) and is activated, thus generating the second messenger 2′3′-cyclic GMP-AMP (cGAMP), which in turn activates membrane-bound stimulator of interferon genes (STING) and induces interferons (IFNs). (B-E) Intercellular transmission of cGAS-STING signaling, including the transfer of dsDNA through phagocytosis, facilitated internalization, and extracellular vesicles (B); the intercellular transmission of cGAMP through gap junctions, receptor-based transport, viral particles, and membrane fusion (C); the intercellular transmission of activated STING triggered by RAB22A-mediated non-canonical autophagy (D); the intercellular communication of IFN signaling through autocrine and paracrine ligand-receptor systems, and subsequent activation of the Janus kinase (JAK)-signal transducer and activator of transcription (STAT) pathway and induction of the expression of various interferon-stimulated genes (ISGs) (E).

## Intercellular transmission of dsDNA

Unlike normal cells, free cytoplasmic dsDNA commonly arises in tumor cells because of chromatin instability and altered metabolism. These dsDNAs are derived from multiple sources, including genomic, mitochondrial, apoptotic, and transposable elements, all of which activate cGAS-STING signaling and subsequently induce innate immune responses^3^. The modes of dsDNA transmission include phagocytosis, facilitated internalization, and extracellular vesicles (EVs).

### Phagocytosis

dsDNA transfer between cancer cells and immune cells typically occurs through phagocytosis in the tumor microenvironment. Tumor cell-derived DNA accumulates in the cytoplasm of phagocytes, such as dendritic cells and macrophages, and triggers the activation of cGAS-STING signaling, thus facilitating antigen presentation and promoting acquired immune responses^4^.

### Facilitated internalization

High levels of dsDNA are released into extracellular space after cell death. This DNA is typically non-immunogenic because of its rapid degradation. However, some antimicrobial peptides, such as LL37, bind extracellular dsDNA and facilitate its internalization into monocytes, which produce type I IFNs^5^.

### Extracellular vesicles

dsDNA associated with small EVs (<200 nm in diameter) has been found to mediate intercellular communication in scenarios including infection, inflammation, and malignancy. EVs are lipid bilayer-bound vesicles in biological fluids and are released and captured by cells. EV-mediated delivery of foreign DNA to bystander cells is frequently detected in pathogen infections^6^. Tumor cell-derived EVs with dsDNA cargo regulate the tumor microenvironment, thereby promoting tumor progression and metastasis. In Crohn’s disease, EVs carrying dsDNA are shed from sites of inflammation and subsequently activate cGAS-STING signaling and enhance innate immune responses in macrophages, thus aggravating the disease^7^. Upregulation of the mitochondrial DNA-binding protein Lon also induces the secretion of EVs with mtDNA and PD-L1, thereby further inducing the production of IFN and IL-6, and attenuating T-cell immunity. Because dsDNA is found in many stages of cancer and has the potential to strengthen cancer immunity and mediate metastasis, further exploration of its generation and transmission is expected to yield exciting findings in cancer biology.

## Intercellular transmission of cGAMP

cGAMP is secreted by cancer cells into the extracellular space and subsequently activates immune responses in receptor cells through various paracrine pathways. Transfer of cGAS-synthesized cGAMP to bystander cells is a primary mechanism underlying cytokine-independent activation of innate and adaptive immune responses. The extracellular hydrolase ENPP1 degrades extracellular cGAMP, thereby controlling the levels of cGAMP in the extracellular environment^8^.

### Gap junctions

Cells transfer cGAMP to neighboring immune cells and activate the immune response through gap junctions, intercellular structures consisting of arrays of intercellular channels in the plasma membrane that allow for the direct transfer of ions and small molecules between cells. Connexins form gap junctions in vertebrates, and transmission of cGAMP relies on the expression of connexins. For instance, connexins 43 and 45 enable the transfer of cGAMP between epithelial and macrophages^9^. This macrophage transactivation amplifies positive-feedback loops of antitumor immune responses. However, cGAMP transfer also promotes tumor growth and metastasis. Brain metastatic cancer cells use gap junctions to transfer cGAMP into astrocytes in a process mediated by protocadherin 7 and connexin 43, thus resulting in the propagation of cytokines that support tumor growth, such as interferon-α (IFN-α) and tumor necrosis factor (TNF)^10^. In this scenario, the modulators of gap junctions that break this paracrine loop may serve as targets to treat established brain metastasis.

### Receptor-based cGAMP transport

Unlike gap junctions, receptor-based transport facilitates the transmission of cGAMP to distant cells. The volume-regulated anion channel (VRAC) mediates cGAMP import and export depending on the cGAMP chemical gradient. Knockdown of leucine-rich repeat containing 8 (LRRC8A) has been found to eliminate VRAC activity and to inhibit 50%–70% of cGAMP uptake in various primary and cultured cells^11^. Other cGAMP transporters, including solute carrier family 19 member 1 (SLC19A1) and SLC46A2, have been found to have roles in cells such as THP-1, Nu-DUL-1, U937, and CD14^+^ monocytes, according to two recent studies^12,13^. ATP-gated P2X purinergic receptor 7 (P2X7R) is another protein that may potentially transport cGAMP^14^. Opening the ATP-gated P2X7R channel requires a high concentration of ATP, which may be present during cell damage or death, thus allowing nanometer-sized molecules such as cGAMP to pass through. Therefore, receptor-based transport of cGAMP requires various yet-to-be-identified transporters and functions in a context-dependent manner.

### Viral particles and membrane fusion

Expressing HIV-1 Env in donor T cells leads to a membrane fusion reaction with uninfected CD4^+^ macrophages. The intercellular transfer of cGAMP through fusion sites augments type I IFN responses in macrophages^15^. HIV-1 also rapidly transfers cGAMP to surrounding cells through viral particles, as validated by mass spectrometry analysis. These viral particles containing cGAMP stimulate robust STING-mediated immune responses in cGAS-deleted dendritic cells^16,17^. However, the role of cGAMP transmission by viral particles and membrane fusion is less understood in cancer biology.

## Intercellular transmission of activated STING proteins

STING is an ER-resident transmembrane protein that traffics to the Golgi after activation, and is degraded through autophagy and lysosomes. The lifespan of STING is associated with the membrane system. Notably, the Kang group^18^ has recently revealed that activated STING proteins are packaged into RAB22A-induced EVs and transported intercellularly through non-canonical autophagy. EVs containing activated STING induce downstream effects of cGAS-STING signaling in recipient cells. Host-derived STING proteins have been detected in exosomes isolated from HSV-1 infected cells, although no secretion signal peptide in STING has been identified. Activated STING proteins are packaged into Rafeesomes, the organelle formed by the fusion of ER-derived and RAB22A-mediated non-canonical autophagosomes with RAB22A-positive early endosomes^18^. During the process, RAB22A inactivates RAB7, thereby suppressing the fusion of the Rafeesome with the lysosome, and enabling the secretion of these inner vesicles containing activated STING as EVs. These STING-containing EVs induce IFN-β release from recipient cells into the tumor microenvironment, thus promoting antitumor immunity. These intriguing observations provide a new perspective regarding the spread of STING-armed immunity and alternative forms of intercellular transmission of functional membrane proteins.

## Intercellular transmission of interferons

IFNs have various functions in immunity and diseases, such as adaptive immune regulation, pathogen defense, antitumor immunity, and autoimmune responses^19^. Type I and III IFNs are crucial secretory proteins downstream of Toll-like receptors, RIG-I-like receptors, and cGAS that activate the JAK-STAT pathway in a paracrine manner by binding receptors of bystander cells such as IFNAR1 and IFNAR2. This ligand-receptor-based intercellular communication of IFNs promotes the generation of hundreds of interferon stimulated genes, thus affecting and modulating IFN signaling^19^. Intercellular transmission of IFN signals in cancer commonly occurs and is crucial for modulating tumorigenesis and cancer progression, such as through directly prolonging the cell cycle and inducing apoptosis. IFNs restrain tumor metastasis by affecting vascular endothelial cells and increasing antitumor immunity in immune cells^3^, such as through activating dendritic cells and cytotoxic CD8^+^ T cells. Consequently, IFN-β is a potential adjuvant for cancer therapy^20^. However, the effects of IFNs on cancer biology are complex, and the protumorigenic effects of IFNs are also well accepted.

## Conclusions

In most scenarios, the activation of cGAS-STING signaling facilitates antitumor immunity; consequently, leveraging the intercellular transmission of cGAS-STING signaling might serve as a promising target for treating cancers. The intercellular transmission of cGAS-STING signaling might be targeted, probably context-dependent, to benefit disease therapeutics (**Table 1**). For example, the physiological concentration of cGAMP is controlled by the cGAMP hydrolase ENPP1, which serves as a potential target for leveraging cGAS-STING transmission. A potent membrane-impermeable ENPP1 inhibitor, compound 32, has been found to enhance plasma cGAMP concentrations and alleviate tumor burden^21^ significantly and has been studied in a clinical trial^22^. Furthermore, DNA-containing EVs secreted from topotecan-treated cancer cells have been found to activate dendritic cells and to facilitate antitumor immunity *via* cGAS-STING signaling. In contrast, pharmacological inhibition of gap junctions hinders the transfer of cGAMP from cancer cells to astrocytes and restricts brain metastasis^10^. The delivery of activated STING *via* EVs adds another layer for leveraging cGAS-STING signaling by inducing the production of IFN-β and facilitating antitumor immunity^18^. However, activators and inhibitors of cGAMP transporters, such as LRRC8A, remain to be developed and examined.

**Table 1:** Clinical trials targeting the intercellular transmission of cGAS-STING signaling in cancers

Elements	Classification	Investigational products	Indications
dsDNA	Extracellular vesicles	Topotecan (U.S. FDA approved)	Lung cancer
cGAMP	ENPP1 inhibitor	RBS2418	Advanced cancer
	Gap junction inhibitor	Tonabersat, carabersat	Brain metastasis
	Transporter activator	Not yet developed	Not yet developed
STING	Extracellular vesicles	Not yet developed	Not yet developed

The beneficial roles of cGAS-STING signaling in cancer progression have recently gained considerable attention. Other critical questions, such as how multiple innate DNA sensors activate the context-dependent response programs and crosstalk with each other and how the cGAS-STING pathway communicates with cancer-specific metabolism and nutrients, are intriguing questions remaining to be answered.
