# Erratum to Intercellular transmission of cGAS-STING signaling in cancer

**DOI:** 10.20892/j.issn.2095-3941.2023.0087

**Published:** 2023-05-04

**Authors:** Qirou Wu, Xiaohong Leng, Pinglong Xu

**Affiliations:** 1 MOE Laboratory of Biosystems Homeostasis and Protection and Zhejiang Provincial Key Laboratory for Cancer Molecular Cell Biology, Life Sciences Institute, Zhejiang University, Hangzhou 310058, China; 2 Institute of Intelligent Medicine, Hangzhou Global Scientific and Technological Innovation Center, Zhejiang University (HIC-ZJU), Hangzhou 310058, China; 3 Cancer Center, Zhejiang University, Hangzhou 310058, China

There are errors in **Figure 1D**^1^ on page 94. Specifically, RAB22A-induced autophagosomes were inadvertently marked in **Figure 1D**. We have revised **Figure 1** to correct the errors. Of note, the errors do not impact the conclusion of this article. We apologize for the errors and for any resulting confusion.

**Figure 1D:**
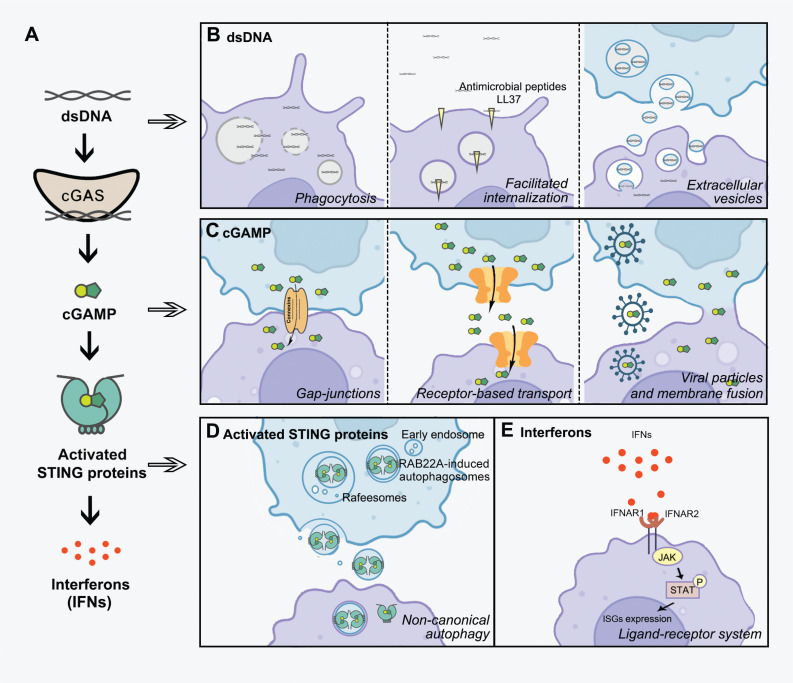
Intercellular transmission of activated STING triggered by RAB22A-mediated non-canonical autophagy.
